# Experience of violence and attitudes of staff members towards coercion in psychiatric settings: observational study

**DOI:** 10.1192/bjo.2024.29

**Published:** 2024-04-15

**Authors:** Alexandre Wullschleger, Anne Chatton, Noémie Kuenzi, Rachel Baeriswyl, Stefan Kaiser, Javier Bartolomei

**Affiliations:** Department of Psychiatry, Geneva University Hospitals, Geneva, Switzerland

**Keywords:** Coercion, attitudes, violence, staff training, in-patient psychiatry

## Abstract

**Background:**

Among important dimensions related to the use of coercive measures, professionals’ attitude towards coercion is of particular interest. Little is known about how experiences of violence in the workplace might influence these attitudes.

**Aims:**

The present study aimed to investigate potential correlates of attitudes towards coercion, especially experiences of violence in the workplace.

**Method:**

Mental health professionals were contacted through an online survey to assess their attitudes towards coercion using the Staff Attitude to Coercion Scale (SACS). The three subscales of the SACS (critical, pragmatic and positive attitudes) were analysed in a multivariate multiple linear regression, using a set of covariates including experiences of violence in the workplace. We hypothesised that experience of violence in the workplace would correlate with less critical attitudes of staff members towards coercion.

**Results:**

A total of 423 professionals were included in the regression analysis. Age, professional category, feeling of insecurity, having witnessed or used coercion, and the emotional burden associated with coercive measures had a joint significant effect on the three SACS subscales. A feeling of insecurity, but not the experience of violence, was associated with a less critical, more positive appraisal of coercive measures. The emotional burden related to the use of coercion was associated with a more critical attitude.

**Conclusions:**

The present results highlight the importance of considering staff members’ training and well-being regarding their feelings of insecurity when addressing attitudes towards coercion. The experience of patients should be integrated into staff training and coercion reduction programmes.

The potential dramatic consequences of coercive measures such as seclusion, restraint and forced medication are well-known.^1^ There is great variation in the use of coercion among countries, regions and institutions that has not been fully explained to date. Factors directly related to staff members have been discussed as potentially influencing the use of coercion,^2^ and professionals’ attitudes towards coercion have been investigated as potential factors that might reflect individual and institutional perceptions of coercive practices.^3–5^

Cultural, ward-related, individual and patient-related factors all seem to influence staff members’ attitudes.^6^ Regarding differences between professional groups, a study reported that psychiatrists and psychologists tend to have more critical, less positive attitudes towards coercion than nurses.^7^ Another study yielded similar results, with psychiatrists, especially senior clinicians, and experienced staff members showing more critical attitudes towards coercion.^8^ An Indian study also found that psychiatrists tended to have a more critical view of coercion compared with patients’ relatives.^9^ Steinert et al also showed that social workers and psychologists tended to approve coercion less than psychiatrists or nurses,^10^ and findings by Vandamme et al similarly supported the hypothesis that nurses were more positive regarding the use of coercion.^11^

Recent work by Krieger et al supported the hypothesis that staff attitudes are directly related to particular emotional aspects linked to their experiences at work.^12^ As an example, high levels of emotional exhaustion are related to more positive appraisal of coercion among nurses.^13^ Most studies show that staff members tend to have a pragmatic view of coercion as necessary for providing care and security.^4,14^ Regarding this aspect, the perceived level of aggression on wards and the impression of insufficient safety measures seem to be associated with the use of coercion.^15^ Nurses’ feeling of safety was also shown to be potentially associated with the use of seclusion in another study.^16^ According to the scientific literature, nurses are among the professional categories most at risk of experiencing violence at work during their career, especially in mental health settings.^17,18^ A history of assault by a patient was found to be associated with more frequent use of mechanical restraint in a study by Moyan and colleagues.^19^ Violence and coercion in psychiatric care are thus highly entangled issues. However, there has not yet been any research investigating the influence of experience by staff members of violence and their attitudes towards coercion.

The present work thus aimed to investigate potential correlates of attitudes of staff members towards coercion, especially their experience of violence in the workplace. We hypothesised that experiences of violence at work would be correlated with less critical, more positive attitudes towards coercion.

## Method

### Participants and procedures

This was an observational, cross-sectional study. Staff members of the Department of Psychiatry of the Geneva University Hospital were contacted and asked to complete a single anonymous online survey (*n* = 881). These included nurses, psychiatrists, psychologists, social workers, occupational and psychomotor therapists, and administrative and cleaning staff.

Study data were collected through an anonymous online questionnaire using the Research Electronic Data Capture (REDCap) tools.^20,21^ The survey included comprehensive information about the study protocol, and participants were asked to give their explicit consent to participation. Data were gathered during an 8 month period.

The authors assert that all procedures contributing to this work comply with the ethical standards of the relevant national and institutional committees on human experimentation and with the Helsinki Declaration of 1975, as revised in 2008. The local ethics committee waived the project because it did not fall under national legislation on human research (*Commission cantonale d’éthique de la recherche*; no. 2021-00303).

### Legal framework of coercive measures in Switzerland

The Swiss Civil Code regulates the use of coercive measures in Switzerland. Measures physically limiting movement such as seclusion or mechanical restraint are only allowed in cases of severe danger to oneself or others, or serious disruption to community life. All decisions to apply a coercive measure are prescribed by medical doctors after the approval of a senior physician. Prescriptions are valid for a 24 h period and must be renewed if necessary. A written decision is handed to the patient or his/her authorised representatives. Patients can appeal against this decision within 10 days.

### Outcomes and instruments

#### Sociodemographic data

We collected data regarding gender, age category, job position, part-time activity, service, work setting (in- or out-patient) and years of experience in psychiatry.

#### Experience of violence in the workplace and feeling of insecurity

Using an *ad hoc* questionnaire developed for the project, the following data were gathered: experience of violent events (physical or verbal assault) during the past 12 months; nature and number of events experienced, number and duration of periods of sick leave following a violent incident during the past 12 months; feeling of insecurity at work, rated on a visual analogue scale ranging from 0 (no insecurity) to 100 (extreme insecurity); quality of support received after violent events, rated on a five-point Likert scale; and completion of violence prevention training in the past 3 years. Participants were also asked whether they had witnessed or used coercive measures (seclusion, restraint, forced medication) during the past 12 months and to rate the emotional burden of such measures on a scale ranging from 0 (no burden) to 100 (extreme burden).

#### Attitude to coercion

Staff attitudes to coercion were assessed with the Staff Attitude to Coercion Scale (SACS).^22^ The SACS comprises 15 items rated using a five-point Likert scale ranging from 1 (strongly disagree) to 5 (strongly agree) and assesses three dimensions of the individual's attitude towards coercion. Critical attitude relates to coercion as offensive towards patients. It includes six items with a score range of 6–30. The higher the score, the more staff perceive coercion as offending patients. Pragmatic attitude views coercion as a means of providing care and safety. It consists of six items and scores between 6 and 30. Higher scores indicate greater appraisal of coercion as necessary for care and security reasons. Positive attitude relates to coercion as treatment and consists of three items (scores 3–15). Here, higher scores indicate a stronger perception of coercion as necessary for treatment. A French translation of the instrument was made for this study using a back-translation method. The original scale showed good psychometric properties.^22^ Internal consistency as assessed for each dimension by Cronbach's alpha coefficient was 0.70 for critical attitude, 0.73 for pragmatic attitude and 0.69 for positive attitude. For our study, these coefficients were 0.78, 0.81 and 0.68, respectively.

#### Symptoms of burnout

To assess the presence and intensity of burnout symptoms, we used the French version of the Oldenburg Burnout Inventory (OLBI).^23,24^ The OLBI is a 16-item self-rating instrument that assesses two core dimensions of burnout, namely exhaustion and disengagement. Each dimension comprises eight items whose ratings range from 1 (strongly agree) to 5 (strongly disagree). Negatively worded items (four in each dimension) are reversed-scored so that higher scores for each item correspond to more negative responses. Hence, higher scores indicate higher levels of exhaustion and disengagement, respectively. The score range of each dimension is from 8 to 32 The French version showed good psychometric properties, with Cronbach's alpha of 0.81 for the exhaustion subscale and 0.69 for the disengagement subscale.^24^ In the current study, the Cronbach's alpha coefficients were 0.86 and 0.73, respectively.

### Statistical analyses

Descriptive statistics such as mean and standard deviation or percentages were computed to summarise participants’ characteristics.

We addressed the research question by fitting a multivariate multiple linear regression, a statistical method used to assess the effects of a set of covariates on more than one dependent variable. The Pillai's trace statistic, known for its robustness to departure from normality and adequate power to detect true differences in a variety of situations, was used to test the hypothesis that the explanatory variables have a significant effect on the dependent variables.^25,26^ The overall null hypothesis test for the multivariate model is *H*_0_: *B* = 0. Its values range from 0 to 1, and increasing values mean that effects are significant, rejecting the null hypothesis that the coefficients are 0. Beforehand, the modelling of separate regressions with the same regressors was deemed necessary. As we had multiple responses, the assumption of multivariate normality of the residuals was also assessed through the Shapiro–Wilk test.

Hence, the three SACS subscale scores (critical, pragmatic and positive attitudes), used as dependent variables, were modelled as functions of age (≤29 years *v.* 30–39 years, *v.* 40–49 years *v.* ≥50 years); gender (female *v.* male); job position (nursing assistant/nurse *v.* medical doctor *v.* occupational therapist/psychologist); experience of violent events during the past 12 months (yes *v.* no); feeling of insecurity, exhaustion or disengagement (OLBI scores); experience with coercion during the past 12 months (yes *v.* no); years of professional experience; work setting (in-patient *v.* out-patient); and emotional burden of coercive measures as covariates. The choice of explanatory variables was determined by previous scientific findings and driven by our main hypothesis.^4^ Categorical variables with *k* categories were transformed into *k* − 1 dummy variables to be considered in the regression, the *k*^th^ being set aside as the reference category against which all other categories were compared. These were younger age, male gender, nursing assistant/nurse position, out-patient setting, absence of a violent event and no use of coercion. When performing multivariate multiple linear regressions, the same covariates and the same reference category must hold during the phase of the multiple regression procedure for the overall multivariate test to be valid.

The core assumptions made by multiple linear regression (normality, linearity, homoscedasticity and independence of residuals) were assessed through plots of residuals in routine pre-analysis screening.^27^ In cases of multicollinearity, any value of the variance inflation factor greater than 5 should be a matter for concern.^28^

### Missing values

Four-hundred and seventy-seven records were extracted from the REDCap platform. After exclusion of those who did not consent to participate, we were left with a sample of 468 respondents, with incomplete data up to 8%. Missingness was dealt with using the expectation-maximisation algorithm, a single-imputation simulation technique which imputes missing values by an iterative procedure. Missingness by design, i.e. when responses are not expected for respondents who do not experience a particular event, was not imputed.

### Sample size estimate

Based on a previous study conducted in the Department of Psychiatry, a response rate of about 30% (or 300 participants) was expected.^29^ Referring to Green, the common rule-of-thumb sample size calculation for univariate multiple regression models is *N* ≥ 50 + 8 *m*, where *m* is the number of predictors.^30^ Adapting this rule for the multivariate case (*N* ≥ 3 × 50 + 8 *m*) led to a minimum sample of 270 people (three dependent variables and 15 common predictors). Of note, the 45 administrative and cleaning staff who were part of the survey were excluded from the regression analyses as they had no direct contact with coercive measures, unlike the health professionals. Thus, the sample of 423 participants was sufficient to address the research questions.

For all analyses, significance was set at *P* < 0.05. We used IBM SPSS for estimation of missing values and R to conduct the multivariate procedures, in particular, the ‘car’ and ‘mvnormtest’ packages.^31,32^

## Results

### Descriptive analysis

Descriptive statistics are summarised in Table 1. Overall, 468 participants responded to the survey (response rate: 53.1%). Participants were predominantly women (63.9%), nursing assistant/nurses (56.6%) and worked in an in-patient setting (64.1%). A majority had used or witnessed coercive measures during the past 12 months (67.5%), 27.1% had experienced physical assault at the workplace during the same period, and 71.6% were victims of verbal assault. Only 34.6% of the participants had received violence prevention training over the past 3 years.

**Table 1:** Characteristics of surveyed participants

Demographic and other characteristics	*N* or median (range)	Percentage or mean ± s.d.	Missing imputed^a^
Age, years			Yes
≤29	55	11.8	
30–39	141	30.1	
40–49	127	27.1	
≥50	145	31.0	
Gender			Yes
Male	169	36.1	
Female	299	63.9	
What is your job?			Yes
Nursing assistant/nurse	265	56.6	
Medical doctor	93	19.9	
Psychologist or occupational therapist	65	13.9	
Administrative, cleaning staff, other	45	9.6	
How many experience years do you have in psychiatry?	11.2 (0–42)	13.5 ± 10.2	Yes
In what healthcare sector do you currently work?			Yes
Out-patient	168	35.9	
In-patient	300	64.1	
In the past 12 months, did you witness or apply yourself coercive measures?			Yes
Yes	316	67.5	
No	152	32.5	
What was the emotional impact of such measures on you?	51.7 (0–100)	51.7 ± 21.3	Yes
In the past 12 months, did you experience a violent event (physical or verbal attack) at your workplace?			Yes
Yes	340	72.6	
No	128	27.4	
In the past 12 months, did you experience physical attacks at your workplace?			Yes
Yes	127	27.1	
No	341	72.9	
In the past 12 months, did you experience verbal attacks at your workplace?			Yes
Yes	335	71.6	
No	133	28.4	
In the past 12 months, how often do you believe you have been a victim of verbal attack?			No
Rarely	62	13.2	
Less than once a month	71	15.2	
Several times a month	124	26.5	
Several times a week	45	9.6	
Every day	16	3.4	
In the past 3 years, did you receive training on violence prevention			No
Yes	162	34.6	
No	285	60.9	
Using a 100 point scale, indicate your feeling of insecurity	30 (0–100)	35.1 ± 26.4	Yes
OLBI disengagement subscore	17 (8–29)	17.4 ± 3.6	Yes
OLBI exhaustion subscore	19 (8–32)	18.9 ± 4.4	Yes
SACS critical attitude subscore	19 (6–30)	19.5 ± 3.9	Yes
SACS pragmatic attitude subscore	23 (6–30)	23.3 ± 3.5	Yes
SACS positive attitude subscore	8 (3–15)	7.7 ± 2.2	Yes

OLBI, Oldenburg Burnout Inventory; SACS, Staff Attitude to Coercion Scale.

a. Only variables in the regression models and auxiliary variables that may have contained information about the missing data were imputed.

### Regression analysis

No violations of key assumptions for conducting multiple regression were detected. There was no multicollinearity between the covariates, as evidenced by the variance inflation factor (no value exceeding 5).

The results of three separate standard multiple linear regressions with (a) critical attitude, (b) pragmatic attitude and (c) positive attitude subscores as dependent variables are given in Table 2. The adjusted R-squared values were 20% for SACS critical attitude, 12% for SACS pragmatic attitude and 14% for SACS positive attitude.

**Table 2:** Multiple regression estimates for SACS critical, pragmatic and positive attitudes

SACS dimension	Parameter	Beta	s.e.	*t*-value	*P*-value
Critical attitude					
	Intercept	17.68	1.26	14.06	<0.001
	Age				
	≤29 years old (ref. cat.)	–	–	–	–
	30–39 years old	−0.86	0.61	−1.41	0.2
	40–49 years old	−2.04	0.70	−2.93	0.004
	≥50 years old	−2.22	0.64	−2.65	0.008
	Gender				
	Female	−0.93	0.38	−2.44	0.01
	Male (ref. cat.)	–	–	–	–
	Job category				
	Nursing assistant/nurse (ref. cat.)	–	–	–	–
	Medical doctor	1.20	0.50	2.40	0.02
	Occupational therapist/psychologist	1.66	0.57	2.93	0.004
	Years professional experience	0.05	0.03	1.77	0.08
	Work setting				
	In-patient	−0.24	0.47	−0.50	0.6
	Out-patient (ref. cat.)	–	–	–	–
	Violent event				
	Yes	−0.46	0.50	−0.92	0.4
	No (ref. cat.)	–	–	–	–
	Feeling of insecurity	−0.03	0.01	−3.24	0.001
	OLBI disengagement	−0.01	0.07	−0.17	0.9
	OLBI exhaustion	−0.02	0.06	−0.26	0.8
	Witnessing or using coercion oneself				
	Yes	1.17	0.52	2.24	0.03
	No (ref. cat.)	–	–	–	–
	Emotional burden of coercive measures	0.08	0.01	8.79	<0.001
Pragmatic attitude					
	Intercept	23.17	1.12	19.80	<0.001
	Age				
	≤29 years old (ref. cat.)	–	–	–	–
	30–39 years old	−0.24	0.57	−0.42	0.7
	40–49 years old	−0.14	0.65	−0.21	0.8
	≥50 years old	−0.12	0.78	−0.15	0.7
	Gender				
	Female	0.15	0.35	0.43	0.7
	Male (ref. cat.)	–	–	–	–
	Job category				
	Nursing assistant/nurse (ref. cat.)	–	–	–	–
	Medical doctor	1.07	0.47	2.29	0.02
	Occupational therapist/psychologist	−0.17	0.53	−0.32	0.7
	Years professional experience	−0.02	0.03	−0.62	0.5
	Work setting				
	In-patient	−0.27	0.44	−0.61	0.5
	Out-patient (ref. cat.)	–	–	–	–
	Violent event				
	Yes	0.68	0.46	1.47	0.1
	No (ref. cat.)	–	–	–	–
	Feeling of insecurity	0.03	0.08	3.66	<0.001
	OLBI disengagement	0.08	0.06	1.26	0.2
	OLBI exhaustion	−0.02	0.06	−0.38	0.7
	Witnessing or using coercion oneself				
	Yes	0.45	0.49	0.93	0.3
	No (ref. cat.)	–	–	–	–
	Emotional burden of coercive measures	−0.05	0.01	−5.81	<0.001
Positive attitude					
	Intercept	7.70	0.72	10.75	<0.001
	Age				
	≤29 years old (ref. cat.)	–	–	–	–
	30–39 years old	−0.44	0.35	−1.25	0.2
	40–49 years old	0.04	0.40	0.11	0.9
	≥50 years old	0.22	0.48	0.47	0.6
	Gender				
	Female	0.19	0.22	0.89	0.4
	Male (ref. cat.)	–	–	–	–
	Job category				
	Nursing assistant/nurse (ref. cat.)	–	–	–	–
	Medical doctor	0.06	0.29	0.20	0.8
	Occupational therapist/psychologist	0.19	0.32	0.58	0.6
	Years professional experience	−0.02	0.02	−1.24	0.2
	Work setting				
	In-patient	−0.14	0.27	−0.54	0.6
	Out-patient (ref. cat.)	–	–	–	
	Violent event				
	Yes	0.34	0.28	1.19	0.2
	No (ref. cat.)	–	–	–	
	Feeling of insecurity	0.01	0.005	2.13	0.03
	OLBI disengagement	0.07	0.04	1.72	0.1
	OLBI exhaustion	−0.003	0.03	−0.08	0.9
	Witnessing or using coercion oneself				
	Yes	−0.50	0.30	−1.0.67	0.1
	No (ref. cat.)	–	–	–	–
	Emotional burden of coercive measures	−0.02	0.005	−5.08	<0.001

SACS, Staff Attitude to Coercion Scale; ref. cat., reference category; OLBI, Oldenburg Burnout Inventory.

### Critical attitude

Considering SACS critical attitude, age, gender, job category, feeling of insecurity, using or witnessing coercion, and emotional burden of coercive measures were significantly associated with the dependent variable. Older age was negatively associated with critical attitudes (*P* = 0.004 and *P* = 0.008 for 40–49 and 50+ years, respectively). In other words, older staff members were less likely to perceive coercion as offending patients compared with the ≤29-year-old category. Similarly, gender and feeling of insecurity were negatively associated with the dependent variable (*P* = 0.01 and *P* = 0.001, respectively). Compared with males, females perceived coercion as less offending. Likewise, staff members showing higher levels of insecurity were less critical towards coercion. Conversely, job category, witnessing or using coercion, and the emotional burden associated with coercive measures were all positively associated with the dependent variable. Compared with nursing assistants/nurses, psychiatrists and occupational therapists/psychologists were more likely to score high on this scale (*P* = 0.02, *P* = 0.004). The same was true for participants having witnessed or used coercion compared with those who did not (*P* = 0.03). Likewise, the level of emotional burden was positively associated with critical attitude scores (*P* < 0.001). Experience of violent events in the past 12 months was not significantly associated with critical attitude subscale scores (*P* = 0.4).

### Pragmatic attitude

Regarding the SACS pragmatic attitude subscale, job category, feeling of insecurity and emotional burden of coercive measures all had significant effects on the dependent variable. Compared with nursing assistants/nurses, being a medical doctor had a positive association with the dependent variable. In other words, medical doctors viewed coercion as necessary for safety and security reasons more than nurses (*P* = 0.02). Higher levels of perceived insecurity were also associated with higher subscale scores (*P* < 0.001). For the emotional burden, a negative association was observed. Reporting of high levels of emotional burden associated with the use of coercive measures was correlated with lower appraisal of coercion as necessary for safety reasons (*P* < 0.001). There was no association with experience of violent events (*P* = 0.1).

### Positive attitude

Regarding SACS positive attitude, feeling of insecurity and emotional burden of coercive measures were the only significant covariates. Indeed, a feeling of insecurity was positively associated with the dependent variable. Higher levels of insecurity were correlated with a stronger perception of coercion as a treatment intervention (*P* = 0.03). On the contrary, the emotional burden of coercive measures was negatively associated with this subscale. Higher experienced emotional burden in relation to the use of coercion was associated with a less positive attitude (*P* < 0.001). There was no difference between nurses and medical doctors, nor between nurses and occupational therapist/psychologists (*P* = 0.8 and *P* = 0.6, respectively). There was also no influence of experience of violent events (*P* = 0.2).

### Multivariate regression

The result of the Shapiro–Wilk test for multivariate normality of the residuals was *W* = 0.92 (*P* < 0.001).

Pillai's multivariate test of significance (*F* = 5.17, *P* < 0.001) showed that age, job category, feeling of insecurity, witnessing or using coercion, and emotional burden related to coercive measures had joint significant effects on the three dependent variables. More precisely, the null hypothesis that the coefficients for age classes 40–49 and ≥50 years, being a medical doctor or occupational therapist/psychologist, feeling of insecurity, witnessing of using coercion oneself, and emotional burden of coercive measures were zero were rejected at *P* < 0.05 (details are shown in Table 3). The experience of violent events had no influence on the three dependent variables (*P* = 0.05).

**Table 3:** Multivariate regression results for SACS subscores

	d.f.	Pillai's trace	*F*	Num d.f.	Den d.f.	*P*- value
Age						
≤29 years old (ref. cat.)	–	–	–	–	–	–
30–39 years old	1	0.01	1.94	3	406	0.12
40–49 years old	1	0.02	3.74	3	406	0.01
≥50 years old	1	0.02	2.95	3	406	0.03
Gender						
Female	1	0.01	2.11	3	406	0.1
Male (ref. cat.)	–	–	–	–	–	–
Job category						
Nursing assistant/nurse (ref. cat.)	–	–	–	–	–	–
Medical doctor	1	0.04	6.15	3	406	0.0004
Occupational therapist/psychologist	1	0.03	4.01	3	406	0.008
Years professional experience	1	0.01	1.21	3	406	0.3
Work setting						
In-patient	1	0.003	0.43	3	406	0.7
Out-patient (ref. cat.)	–	–	–	–	–	–
Violent event						
Yes	1	0.01	0.84	3	406	0.5
No (ref. cat.)	–	–	–	–	–	–
Feeling of insecurity	1	0.04	5.66	3	406	0.0008
OLBI disengagement	1	0.01	1.24	3	406	0.3
OLBI exhaustion	1	0.001	0.12	3	406	0.9
Witnessing or using coercion oneself						
Yes	1	0.03	4.04	3	406	0.007
No (ref. cat.)	–	–	–	–	–	–
Emotional burden of coercive measures	1	0.17	28.00	3	406	<0.001

SACS, Staff Attitude to Coercion Scale; Den, denominator; Num, numerator; OLBI, Oldenburg Burnout Inventory; ref. cat., reference category.

### Sensitivity analysis

To rule out potential biases induced by including professionals not directly involved in the decision-making processes related to coercive measures, we performed a sensitivity including only nurses and psychiatrists as professional categories. The results of the multivariate regression analysis did not differ from those of the main analysis, except with respect to age. Indeed, job position, feeling of insecurity, witnessing or using coercion and emotional burden of coercive measures had joint significant (*P* < 0.05) effects on the three dependent variables.

## Discussion

The present work investigated the determinants of the attitudes of staff members towards coercion, especially regarding the experience of violent events. The main hypothesis was that the experience of violence in the workplace would be related to a less critical attitude towards coercion.

Our results invalidate this hypothesis, as the experience of violent events in the workplace was not associated with attitudes towards coercion. However, our analysis showed that a feeling of insecurity was significantly associated with a less critical, more positive attitude. This discrepancy seems to indicate that attitudes towards coercion might be influenced not by the experience of violence but rather by the ability to handle the potential for violence and feelings of insecurity. This decisive role of insecurity confirms previous scientific findings.^14,33,34^ It can be hypothesised that feeling unsafe or not in the capacity of handling violent situations might reinforce the vision of coercion as an important, positive means of protection and care. Staff members night thus use coercive measures as means of dealing with the apprehension of violence, while minimising their deleterious consequences. The fact that about two-thirds of our sample had not received any type of violence management training in the past 3 years should be regarded in the light of these results as problematic. As the literature has shown, effective and regular staff training could help to prevent the use of coercive measures and positively influence attitudes towards coercion.^4,35,36^ Such training should provide staff members with de-escalation techniques and alternatives to coercive measures. Special institutional attention should also be paid to support of staff members regarding their feelings of safety, including regular clinical team supervision.

Our results also show that psychiatrists and psychologists/occupational therapists have more critical attitudes towards coercion than nursing staff. This confirms previous literature findings.^10,12^ These results should be carefully considered and discussed. There are major differences between professions in terms of responsibilities regarding coercive measures, as psychiatrists hold the power to decide to use such measures, even if the situation is discussed with other professionals. Psychologists and occupational therapists are normally not directly involved in the decision-making process but rather witness coercive measures being used. It can thus be hypothesised that the influence of job position on attitudes towards coercion is mediated by the role of professionals regarding coercive measures.

As Morandi et al have shown, psychiatrists tend to experience more external pressures (issues that drive the use of coercive measures) and internal pressures (ethical conflicts).^8^ Differences are also notable in the daily activities of health professionals regarding patient contact, with nursing staff engaging in much closer and intensive contact with patients in hospital. The frequency and intensity of these contacts might thus affect the experience of insecurity or the appraisal of a patient's clinical state, in turn influencing one's appraisal of coercion.

Having used or witnessed a coercive measure during the past 12 months was associated with a more critical attitude. This finding should be carefully interpreted as it seems to be in contradiction with previous findings.^8,37–39^ However, most of these previous studies used instruments and scales other than the SACS, limiting the comparability of results. A study by Molewijk et al using the SACS showed that staff members who experienced coercion at least weekly were more likely to have a pragmatic attitude compared with those who never or rarely experienced it.^39^ Regarding interpretation of our finding, it must be mentioned that our Department has been progressively implementing a broad coercion reduction programme over the past years. Thus, staff members were probably already influenced in their perceptions of the more negative aspects of coercive interventions. Furthermore, although a large proportion of staff members took part in the study, we cannot exclude an influence of selection bias, as the staff members most engaged in the matter of coercion reduction could have been more likely to answer the survey. Further qualitative distinctions should also be made in future research to understand potential differences between using or witnessing coercive measures regarding staff members’ attitudes. As discussed above, these differences might reflect different intensities and frequencies of patient contact. The frequency of experiences with coercion and more or less active roles in decision-making could also be interesting variables for a future analysis.

Notably, the emotional burden associated with the use of coercive measures was associated with less positive, more critical attitudes towards coercion, a result in line with previous findings by Krieger and colleagues, who completed the SACS by adding new items to assess staff members’ views and emotions about coercion.^12^ As the SACS assesses mostly cognitive aspects of attitudes towards coercion, assessment of the emotional burden associated with coercion sheds important light on the determinants of these attitudes.^40^ This emotional burden might be linked to past professional or personal experiences and reflect internal conflicts or perceptions directly related to the way patients experience coercion, often marked by feelings of humiliation, shame and dehumanisation.

The integration of patients’ perspectives into staff training regarding the management of violence and the use of coercion could be, in this context, a possible way to influence staff's views about coercive measures. The post-coercion review could prove to be an essential instrument to yield this perspective.^41^ The systematic use of advance statements could also be an important way of carrying the experiences of patients, increasing staff members’ awareness of the consequences of coercive measures and identifying possible alternatives. Furthermore, the involvement of peer workers, whose key role in the facilitation of advanced directives has recently been shown, should be reinforced.^42^

With respect to other influencing factors, our results indicate that gender and age are significantly associated with critical attitudes towards coercion. Past research has yielded mixed and partly contradictory results regarding the role of gender.^43,44^ In our sample, women tended to show slightly less critical attitudes towards the use of coercion. A possible explanation could be that women might perceive insecurity and the risk of violence differently from men and thus be more likely to validate the use of coercion as a means of protection. Qualitative analyses would be very helpful to understand better this association in our context. Similarly, the role of age could not clearly be identified in the literature. Older participants in our sample tended to have less critical attitudes towards coercion. The evolution of psychiatric practices, as well as nursing and medical education, with a stronger focus on issues related to coercion and patients’ rights could explain the tendency of younger, newly trained staff members to be more critical towards coercive practices that might have been more accepted in the past. Younger staff members might thus be more inclined to question coercive practices that do not match their personal ideal representation of their work and to perceive their negative consequences in a more acute way.

Regarding limitations, it must be considered that attitudes towards coercion represent a complex phenomenon linked not only to staff-related variables but also to institutional and organisational factors that were not investigated in the present survey. Existing policies, institutional culture, and architectural and environmental factors all influence attitudes towards coercion.^14^ This wide range of potential influencing factors probably explains the somehow low R-squared values of the regressions. The SACS surely also only captures some aspects of attitudes towards coercion, as acknowledged by the authors of the scale.^3^ To the best of our knowledge, the SACS is the only evaluated instrument for assessment of staff attitudes towards coercion. Newer instruments capturing a broader spectrum of dimensions related to attitudes towards coercion are needed to investigate further this outcome. Social desirability might also have influenced the results, considering the efforts made over the past years to address the issue of coercion in the Department. Last, the relationship between attitude towards coercion and the use of coercive measures remains an unresolved question that could not be addressed in the present study.

However, the fact that the regression models included staff members not involved in decisions relating to coercive measures or in the application of such measures may have biased the results. We chose to include staff members not directly involved in decision-making processes surrounding coercion, as their views might reflect a general institutional perception of coercion. As these professionals are also exposed to violence, it seemed important to include their perceptions in the analysis. Furthermore, most studies investigating attitudes towards coercion also included all professional categories in contact with patients.^12,22,39^ The results of the sensitivity analysis we performed did not differ greatly from those of the main analysis, showing that the inclusion of these groups of professionals did not influence the core results.

Another strength of this work is the large, representative sample of staff members working at our Department, spanning across all professional categories. This large sample, as well as the wide range of investigated variables allowed us to produce important results shedding new light on the issue of the attitude towards coercion.

In conclusion, our results indicate that experience of violent events at the workplace does not influence staff members’ attitudes towards coercion. However, feeling of insecurity and the burden of coercive measures both, although in opposite directions, influence the way staff members perceive coercive measures. Even if the link between attitude and use of coercion remains unclear, institutional coercion reduction policies should focus on changing the perception of coercion by staff members, as their perspectives probably have an impact on the way coercion is applied in psychiatric services. Such coercion reduction programmes should include thorough and regular staff training, including post-coercion review techniques. These training programmes should also have a strong focus on the lived experience of patients who have faced coercive measures.

## Data Availability

The data that support the findings of this study are available from the corresponding author, A.W., upon reasonable request.

## References

[1] Chieze M, Hurst S, Kaiser S, Sentissi O. Effects of seclusion and restraint in adult psychiatry: a systematic review. Front Psychiatry 2019; 10: 491. 31404294 10.3389/fpsyt.2019.00491 PMC6673758

[2] Zinkler M, Priebe S. Detention of the mentally ill in Europe – a review. Acta Psychiatr Scand 2002; 106(1): 3–8. 12100342 10.1034/j.1600-0447.2002.02268.x

[3] Husum TL, Bjørngaard JH, Finset A, Ruud T. A cross-sectional prospective study of seclusion, restraint and involuntary medication in acute psychiatric wards: patient, staff and ward characteristics. BMC Health Serv Res 2010; 10(1): 89. 20370928 10.1186/1472-6963-10-89 PMC2858144

[4] Husum TL, Siqveland J, Ruud T, Lickiewicz J. Systematic literature review of the use of Staff Attitudes to Coercion Scale (SACS). Front Psychiatry 2023; 14: 100. 10.3389/fpsyt.2023.1063276 PMC9941667 36824675

[5] Efkemann SA, Scholten M, Bottlender R, Juckel G, Gather J. Influence of mental health professionals’ attitudes and personality traits on decision-making around coercion: results from an experimental quantitative survey using case vignettes. Acta Psychiatr Scand 2022; 146(2): 151–64. 35322402 10.1111/acps.13429

[6] Happell B, Koehn S. Attitudes to the use of seclusion: has contemporary mental health policy made a difference? J Clin Nurs 2010; 19(21–22): 3208–17. 21040022 10.1111/j.1365-2702.2010.03286.x

[7] Lickiewicz J, Husum TL, Ruud T, Siqveland J, Musiał Z, Makara-Studzińska M. Measuring staff attitudes to coercion in Poland. Front Psychiatry 2021; 12: 745215. 34867536 10.3389/fpsyt.2021.745215 PMC8635088

[8] Morandi S, Silva B, Mendez Rubio M, Bonsack C, Golay P. Mental health professionals’ feelings and attitudes towards coercion. Int J Law Psychiatry 2021; 74: 101665. 33401095 10.1016/j.ijlp.2020.101665

[9] Raveesh B, Pathare S, Noorthoorn EO, Gowda GS, Lepping P, Bunders-Aelen J. Staff and caregiver attitude to coercion in India. Indian J Psychiatry 2016; 58(Suppl 2): S221. 28216773 10.4103/0019-5545.196847 PMC5282619

[10] Steinert T, Lepping P, Baranyai R, Hoffmann M, Leherr H. Compulsory admission and treatment in schizophrenia. Soc Psychiatry Psychiatr Epidemiol 2005; 40(8): 635–41. 16133746 10.1007/s00127-005-0929-7

[11] Vandamme A, Wullschleger A, Garbe A, Cole C, Heinz A, Bermpohl F, et al. The role of implicit and explicit staff attitudes in the use of coercive measures in psychiatry. Front Psychiatry 2021; 12: 699446. 34220595 10.3389/fpsyt.2021.699446 PMC8249742

[12] Krieger E, Moritz S, Lincoln TM, Fischer R, Nagel M. Coercion in psychiatry: a cross-sectional study on staff views and emotions. J Psychiatr Ment Health Nurs 2021; 28(2): 149–62. 32348607 10.1111/jpm.12643

[13] Happell B, Koehn S. Seclusion as a necessary intervention: the relationship between burnout, job satisfaction and therapeutic optimism and justification for the use of seclusion. J Adv Nurs 2011; 67(6): 1222–31. 21261695 10.1111/j.1365-2648.2010.05570.x

[14] Doedens P, Vermeulen J, Boyette LL, Latour C, de Haan L. Influence of nursing staff attitudes and characteristics on the use of coercive measures in acute mental health services – a systematic review. J Psychiatr Ment Health Nurs 2020; 27(4): 446–59. 31876970 10.1111/jpm.12586 PMC7508163

[15] De Benedictis L, Dumais A, Sieu N, Mailhot M-P, Létourneau G, Tran M-AM, et al. Staff perceptions and organizational factors as predictors of seclusion and restraint on psychiatric wards. Psychiatr Serv 2011; 62(5): 484–91. 21532073 10.1176/ps.62.5.pss6205_0484

[16] Vollema MG, Hollants SJ, Severs CJ, Hondius AJK. [Determinants of seclusion in a psychiatric institution: a naturalistic and exploratory study]. Tijdschr Psychiatr 2012; 54(3): 211–21. 22422413

[17] Rasmussen CA, Hogh A, Andersen LP. Threats and physical violence in the workplace: a comparative study of four areas of human service work. J Interpers Violence 2013; 28(13): 2749–69. 23677967 10.1177/0886260513487987

[18] Bilici R, Sercan M, Izci F. Levels of the staff's exposure to violence at locked psychiatric clinics: a comparison by occupational groups. Issues Ment Health Nurs 2016; 37(7): 501–6. 27104294 10.3109/01612840.2016.1162883

[19] Moylan LB, Cullinan M. Frequency of assault and severity of injury of psychiatric nurses in relation to the nurses’ decision to restrain. J Psychiatr Ment Health Nurs 2011; 18(6): 526–34. 21749559 10.1111/j.1365-2850.2011.01699.x

[20] Harris PA, Taylor R, Thielke R, Payne J, Gonzalez N, Conde JG. Research electronic data capture (REDCap) – a metadata-driven methodology and workflow process for providing translational research informatics support. J Biomed Inform 2009; 42(2): 377–81. 18929686 10.1016/j.jbi.2008.08.010 PMC2700030

[21] Harris PA, Taylor R, Minor BL, Elliott V, Fernandez M, O'Neal L, et al. The REDCap consortium: building an international community of software platform partners. J Biomed Inform 2019; 95: 103208. 31078660 10.1016/j.jbi.2019.103208 PMC7254481

[22] Husum TL, Finset A, Ruud T. The Staff Attitude to Coercion Scale (SACS): reliability, validity and feasibility. Int J Law Psychiatry 2008; 31(5): 417–22. 18817973 10.1016/j.ijlp.2008.08.002

[23] Demerouti E, Bakker AB, Vardakou I, Kantas A. The convergent validity of two burnout instruments: a multitrait-multimethod analysis. Eur J Psychol Assess 2003; 19(1): 12.

[24] Chevrier N. Adaptation Québécoise de L'Oldenberg Burnout Inventory (OLBI). Université du Québec à Montréal, 2009.

[25] Olson CL. Comparative robustness of six tests in multivariate analysis of variance. J Am Stat Assoc 1974; 69(348): 894–908.

[26] Pituch KA, Stevens JP. Applied Multivariate Statistics for the Social Sciences: Analyses with SAS and IBM's SPSS. Routledge, 2015.

[27] Tabachnick BG, Fidell LS, Ullman JB. Using Multivariate Statistics. Pearson, 2007.

[28] Sheather S. A Modern Approach to Regression with R. Springer Science & Business Media, 2009.

[29] Bartolomei J, Baeriswyl-Cottin R, Framorando D, Kasina F, Premand N, Eytan A, et al. What are the barriers to access to mental healthcare and the primary needs of asylum seekers? A survey of mental health caregivers and primary care workers. BMC Psychiatry 2016; 16(1): 336. 10.1186/s12888-016-1048-6 PMC5041539 27686067

[30] Green SB. How many subjects does it take to do a regression analysis. Multivar Behav Res 1991; 26(3): 499–510. 10.1207/s15327906mbr2603_7 26776715

[31] IBM Corp. IBM SPSS Statistics for Windows, Version 25.0. IBM Corp, 2017.

[32] R Core Team. R: A Language and Environment for Statistical Computing. R Foundation for Statistical Computing, 2008.

[33] Cusack P, McAndrew S, Cusack F, Warne T. Restraining good practice: reviewing evidence of the effects of restraint from the perspective of service users and mental health professionals in the United Kingdom (UK). Int J Law Psychiatry 2016; 46: 20–6. 27067763 10.1016/j.ijlp.2016.02.023

[34] Goulet M-H, Larue C. A case study: seclusion and restraint in psychiatric care. Clin Nurs Res 2017; 27(7): 853–70. 28608713 10.1177/1054773817713177

[35] Needham I, Abderhalden C, Halfens RJ, Dassen T, Haug HJ, Fischer JE. The effect of a training course in aggression management on mental health nurses’ perceptions of aggression: a cluster randomised controlled trial. Int J Nurs Stud 2005; 42(6): 649–55. 15982464 10.1016/j.ijnurstu.2004.10.003

[36] Price O, Baker J, Bee P, Lovell K. Learning and performance outcomes of mental health staff training in de-escalation techniques for the management of violence and aggression. Br J Psychiatry 2015; 206(6): 447–55. 26034178 10.1192/bjp.bp.114.144576

[37] van Doeselaar M, Sleegers P, Hutschemaekers G. Professionals’ attitudes toward reducing restraint: the case of seclusion in The Netherlands. Psychiatr Q 2008; 79(2): 97–109. 18172765 10.1007/s11126-007-9063-x

[38] Dahan S, Levi G, Behrbalk P, Bronstein I, Hirschmann S, Lev-Ran S. The impact of ‘being there’: psychiatric staff attitudes on the use of restraint. Psychiatr Q 2018; 89(1): 191–9. 28721655 10.1007/s11126-017-9524-9

[39] Molewijk B, Kok A, Husum T, Pedersen R, Aasland O. Staff's normative attitudes towards coercion: the role of moral doubt and professional context–a cross-sectional survey study. BMC Med Ethics 2017; 18(1): 37. 28545519 10.1186/s12910-017-0190-0 PMC5445484

[40] Efkemann SA, Scholten M, Bottlender R, Juckel G, Gather J. A German version of the staff attitude to coercion scale. Development and empirical validation. Front Psychiatry 2021; 11: 573240. 33536947 10.3389/fpsyt.2020.573240 PMC7847975

[41] Wullschleger A, Vandamme A, Mielau J, Stoll L, Heinz A, Bermpohl F, et al. Effect of standardized post-coercion review on subjective coercion: results of a randomized-controlled trial. Eur Psychiatry 2021; 64(1): e78. 10.1192/j.eurpsy.2021.2256 PMC8715283 34872630

[42] Tinland A, Loubière S, Mougeot F, Jouet E, Pontier M, Baumstarck K, et al. Effect of psychiatric advance directives facilitated by peer workers on compulsory admission among people with mental illness: a randomized clinical trial. JAMA Psychiatry 2022; 79(8): 752–9. 35662314 10.1001/jamapsychiatry.2022.1627 PMC9171654

[43] Husum TL, Bjørngaard JH, Finset A, Ruud T. Staff attitudes and thoughts about the use of coercion in acute psychiatric wards. Soc Psychiatry Psychiatr Epidemiol 2011; 46(9): 893–901. 20596695 10.1007/s00127-010-0259-2

[44] Wynn R. Staff's attitudes to the use of restraint and seclusion in a Norwegian university psychiatric hospital. Nord J Psychiatry 2003; 57(6): 453–9. 14630551 10.1080/08039480310003470

